# Determinants of losses in the latent tuberculosis infection cascade of care in Brazil

**DOI:** 10.1136/bmjgh-2021-005969

**Published:** 2021-09-09

**Authors:** Alexandra Brito Souza, María B Arriaga, Gustavo Amorim, Mariana Araújo-Pereira, Betânia M F Nogueira, Artur T L Queiroz, Marina C Figueiredo, Michael S Rocha, Aline Benjamin, Adriana S R Moreira, Jamile G Oliveira, Valeria Rolla, Betina Durovni, José R Lapa e Silva, Afrânio L Kritski, Solange Cavalcante, Timothy Sterling, Bruno B Andrade, Marcelo Cordeiro-Santos, RePORT Brazil consortium

**Affiliations:** 1 Fundação de Medicina Tropical Doutor Heitor Vieira Dourado, Manaus, AM, Brazil; 2 Programa de Pós-Graduação em Medicina Tropical, Universidade do Estado do Amazonas, Manaus, Manaus, AM, Brazil; 3 Laboratório de Inflamação e Biomarcadores, Instituto Gonçalo Moniz, Fundação Oswaldo Cruz, Salvador, BA, Brazil; 4 Multinational Organization Network Sponsoring Translational and Epidemiological Research (MONSTER) Initiative, Salvador, BA, Brazil; 5 Faculdade de Medicina, Universidade Federal da Bahia, Salvador, BA, Brazil; 6 Department of Biostatistics, Vanderbilt University Medical Center, Nashville, TN, USA; 7 Instituto Brasileiro para Investigação da Tuberculose, Fundação José Silveira, Salvador, BA, Brazil; 8 Center of Data and Knowledge Integration for Health (CIDACS), Instituto Gonçalo Moniz, Fundação Oswaldo Cruz, Salvador, BA, Brazil; 9 Division of Infectious Diseases, Department of Medicine, Vanderbilt University School of Medicine, Nashville, TN, USA; 10 Instituto Nacional de Infectologia Evandro Chagas, Fiocruz, Rio de Janeiro, RJ, Brazil; 11 Programa Acadêmico de Tuberculose, Faculdade de Medicina, Universidade Federal do Rio de Janeiro, Rio de Janeiro, RJ, Brazil; 12 Secretaria Municipal de Saúde do Rio de Janeiro, Rio de Janeiro, Brazil; 13 Faculdade de Medicina, Universidade Nilton Lins, Manaus, AM, Brazil

**Keywords:** tuberculosis, cohort study, public health

## Abstract

**Introduction:**

Factors associated with losses in the latent tuberculosis infection (LTBI) cascade of care in contacts of patients with tuberculosis (TB) were investigated in a multicentre prospective cohort from highly endemic regions in Brazil.

**Methods:**

Close contacts of 1187 patients with culture-confirmed pulmonary TB were prospectively studied between 2015 and 2019, with follow-up of 6–24 months. Data on TB screening by clinical investigation, radiographic examination and interferon-gamma release assay (IGRA) were collected. Multivariable regressions were used to identify determinants of losses in the LTBI cascade.

**Results:**

Among 4145 TB contacts initially identified, 1901 were examined (54% loss). Among those examined, 933 were people living with HIV, ≤5 years old and/or had positive IGRA results, and therefore had a recommendation to start TB preventive treatment (TPT). Of those, 454 (23%) initiated treatment, and 247 (54% of those initiating; 26% of those in whom treatment was recommended) completed TPT. Multivariable regression analysis revealed that living with HIV, illiteracy and black/*pardo* (brown) race were independently associated with losses in the cascade.

**Conclusion:**

There were losses at all LTBI cascade stages, but particularly at the initial screening and examination steps. Close contacts of low socioeconomic status and living with HIV were at heightened risk of not completing the LTBI cascade of care in Brazil.

**Key questions**

**What is already known?**

- The treatment of latent tuberculosis infection (LTBI) is a cornerstone for tuberculosis (TB) control and elimination.
- Recent studies indicate that important losses occur at all stages of the LTBI cascade of care. However, the reasons for these losses are not well known.

**What are the new findings?**

- In a large prospective cohort of contacts of persons with TB in Brazil, we identified losses in all LTBI cascade of care stages, mostly at the initial screening and examination steps.
- Low socioeconomic status and HIV infection were significant determinants of losses in the LTBI cascade of care, independent of other confounding factors.

**What do the new findings imply?**

- The greatest losses in the LTBI cascade occurred in people at the highest risk of developing active TB.
- Interventions to improve retention of these groups in the LTBI cascade are urgently needed.

## Introduction

The United Nations (UN) and the WHO have set ambitious targets for reducing the global burden of tuberculosis (TB) by 2030, and recognise the essential role of treatment of latent TB infection (LTBI) as a strategy for TB control and elimination.^1 2^ Management of LTBI involves multiple stages in the care process, from the identification of at-risk populations for testing until completion of TB preventive therapy (TPT).^1 2^

Brazil is one of the 30 high TB-burden nations. Despite strategies implemented by the National TB Control Program, TB rates have changed little in recent years.^3^ Among the most recent recommendations of the Brazilian Ministry of Health was TPT for all TB contacts with a positive interferon-gamma release assay (IGRA) or tuberculin skin testing (TST), as well as all close contacts who are children≤5 years old or people living with HIV (PLWH), regardless of the test result.^4^

Recent studies conducted in high-income and low-income and middle-income countries indicate that important losses occur at all stages of the LTBI cascade of care.^5^ A study conducted in 12 health facilities in three Brazilian cities with high TB incidence rates found that most losses in the cascade occurred in the first two steps, which were contact identification and TST.^6^ Another study found that few people who had a positive TST were started on TPT, and the completion rate was also low.^7^ In a study carried out in a paediatric hospital from Rio de Janeiro, Brazil, there was an association between low human development index and loss to follow-up during TPT in children and adolescents.^8^

Modelling studies suggest that diagnosing and treating LTBI in persons at high risk of developing active disease will accelerate TB elimination.^9 10^ However, there can be many challenges in implementing this policy programmatically. Thus, evaluating the cascade of care of LTBI and the factors associated with failures at each step can provide important insights for TB control.

To date, most of the studies evaluating the LTBI care cascade have been retrospective or cross sectional. Therefore, the established literature does not currently provide robust identification of reliable factors that directly lead to losses in the LTBI care cascade. The current study was performed to fill this important gap in knowledge, and to help design decision-making strategies to improve TB control. We evaluated the LTBI care cascade in a prospective Brazilian cohort of contacts of patients with culture-confirmed pulmonary TB (PTB), and identified factors associated with losses at each phase of the cascade.

## Methods

### Study design

In this study, we included data from close TB contacts identified in the Regional Prospective Observational Research in Tuberculosis (RePORT)-Brazil cohort^11^ between 27 August 2015, and 18 July 2019, with follow-up of up to 24 months, for initiation of TPT, and completion of TPT after initiation.

The RePORT-Brazil consortium is an ongoing, multicentre, cohort study, which follows culture-confirmed PTB cases and their close contacts. Enrolment sites are in three Brazilian states, recruiting participants from five health units: Instituto Nacional de Infectologia Evandro Chagas, Clínica da Familia Rinaldo Delamare and Secretaria de Saúde de Duque de Caxias (all from Rio de Janeiro state), Instituto Brasileiro para Investigação da Tuberculose (Salvador, Bahia), and Fundação Medicina Tropical Dr. Heitor Vieira Dourado (Manaus, Amazonas). The representativeness and operational indicators of the RePORT-Brazil cohort compared with all patients with TB in Brazil have been described previously.^11^ Site details are presented in the online supplemental material 1.

10.1136/bmjgh-2021-005969.supp2 Supplementary data

For the present study, contacts were eligible to participate if exposed to an index case of culture-confirmed PTB who enrolled into RePORT-Brazil and had no evidence of active TB. Exposure to the index TB case was defined as at least 4 hours in 1 week in the 6 months prior to TB diagnosis.

### Procedures

After enrolment of the index case, close contacts were invited to be interviewed and examined at the RePORT-Brazil healthcare units by phone call, text message or in person. Eligible contacts who attended the study sites were approached by study personnel to enrol into the RePORT-Brazil cohort and to be investigated for LTBI. Contacts were evaluated in-person at baseline and 6 months after enrolment; subsequent evaluations every 6 months were by telephone. At baseline, we performed a clinical evaluation, chest X-ray, and collected blood for IGRA and HIV testing. Clinical and demographic data were collected via standardised case report forms. All procedures were performed according to the Brazilian National TB Guideline.^4^ Per the RePORT-Brazil protocol, all contacts returned after 6 months/complete TPT for a new clinical evaluation. Contacts with a negative or indeterminate baseline IGRA test underwent repeat testing at month 6. IGRA collection, processing and interpretation were performed according to the manufacturer’s recommendations for QuantiFERON assay (Qiagen).

We considered eligible for TPT all TB contacts with a positive IGRA, as well as all close contacts who were children≤5 years old or PLWH, regardless of the test result.^2 4^ Isoniazid (6–9 months) were used for TPT,^2 4^ according to the routine practice of the health units and medical provider decision.

All contacts identified by patients with TB and with whom the study team was able to communicate were encouraged to be evaluated clinically and to initiate and complete TPT (if recommended and initiated), regardless of their follow-up in the study.

### LTBI cascade: definitions of each stage of care

We used the cascade of care model published previously^5 7 12^; this model was first used for people with HIV.^13^ The accumulated quantification allowed us to observe the impact of the losses at each stage of care. For this model, the following stages of the LTBI cascade of care were considered: (1) identified by patients with TB as close contacts (reference population); (2) initially screened for LTBI presented to clinic; (3) agreed to participate, signed consent, completed the medical examination, had IGRA performed and radiographic evaluations; (4) recommended to receive TPT; (5) accepted and initiated TPT; (6) completed TPT (defined as:>6 months of isoniazid).

### Definition of losses in the LTBI cascade

For this study, we considered losses in the LTBI cascade among the participants who provided informed consent, were examined and performed the first IGRA, and in addition: (1) contacts who did not perform the second IGRA (when applicable) or (2) did not initiate recommended TPT or (3) did not complete TPT.

### Data analysis

Categorical variables were presented as frequencies and compared using Fisher’s exact test (2×2) or Pearson’s χ^2^ test. Continuous variables were described using median and IQRs and compared using the Mann-Whitney U test (2 groups) or the Kruskal Wallis test (>2 groups).

A multivariable generalised estimating equation^14^ with a logit-link and an independent working correlation matrix was used to identify factors associated with losses in the LTBI cascade of care. Close contacts from the same TB index case and study site were treated as a cluster because participants from the same site could be correlated, as well as close contacts of the same TB index case. Variables included (age, alcohol consumption, comorbidities, illicit drug use, education, HIV infection, income, sex, smoking, secondary smoking, race and time between the baseline visit of the TB case and baseline visit of the contact) in all multivariable regressions were selected a priori, based on clinical factors and the literature.^5 15^

Because a proportion of the data were missing (maximum of 3.3% for income), multiple imputation by chained equations^16^ was used to generate 20 imputed datasets and final estimates were obtained via Rubin’s rule.^17^ The imputation procedure was based on logistic models for categorical variables (race, income and education level), adjusting for baseline variables and outcome. Hierarchical cluster analysis (Ward’s method) was employed to depict the overall profile of the study subgroups stratified according to the final IGRA status.

Data analysis was performed using SPSS V.25.0 (IBM) GraphPad Prism V.8.0 (GraphPad Software, La Jolla, California), JMP Pro V.14.0 (SAS), and R V.3.5.0 (R Foundation). All analyses were prespecified and two tailed. Differences with p value<0.05 were considered statistically significant.

### Patient and public involvement

Neither patients nor the public were involved in the design, conduct or reporting of the research.

## Results

### Characteristics of the study participants

**Figure 1:**
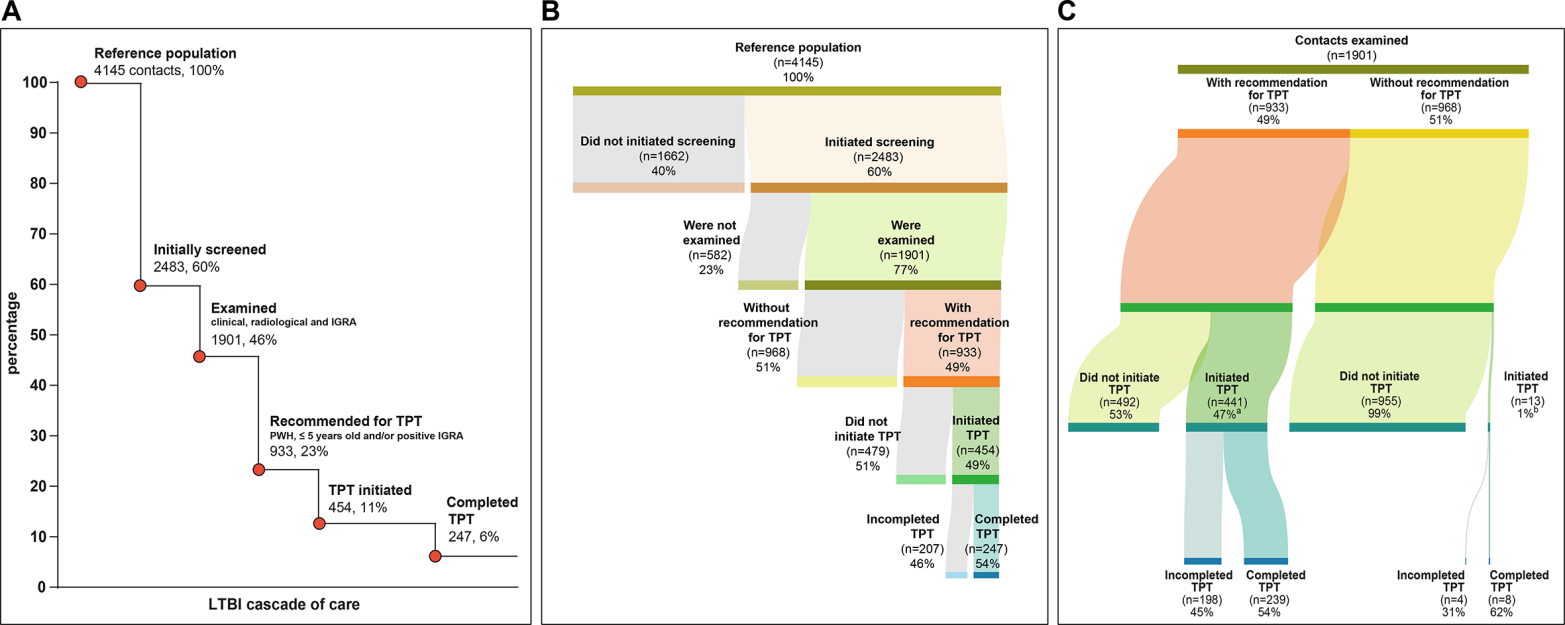
Cascade of care of latent tuberculosis infection (LTBI) in contacts from tuberculosis (TB) cases. (A) Losses and drop-outs at each stage of the LTBI cascade of care. Percentages were calculated among the number of contacts initially identified (n=4145). (B) Reference population and distribution among losses in the LTBI cascade of care and those who completed the LTBI cascade. (C) This figure shows the number of contacts who started treatment and those who completed treatment according to the category of TB preventive therapy (TPT) recommendation. (A) Four contacts were still undergoing TPT at the time of analysis (not considered losses). (B) One contact was still undergoing TPT at the time of analysis (not considered loss). IGRA, interferon-gamma release assay; PWH, people with HIV.

There were 4145 close contacts referred from 1187 PTB cases (average of 3.5 contacts per TB case), but only 2483 contacts of 641 culture confirmed PTB cases could be reached for invitation to present at the healthcare units and showed up for LTBI screening (figure 1A). Of the 2483 contacts who presented to clinic and were invited to participate in the study, 1901 (77%) agreed to participate in the study, provided informed consent and were investigated for LTBI (figure 1A).

The characteristics of TB contacts are presented in table 1. Median age was 32 years (IQR 16.2–46.8), 1130 (59%) were female, 1507 (79%) were black/*pardo* (brown), 192 (10%) were illiterate and 46 (2%) were PLWH. A positive IGRA result was detected in 43% of the study participants, including results from both baseline and month 6. Participants with a positive IGRA were more likely to report smoking (30% vs 24%; p=0.002), and secondary smoking (35% vs 30%; p=0.04) than persons with a negative IGRA result.

**Table 1:** Characteristics of the tuberculosis (TB) contacts by interferon-gamma release assay (IGRA) result

Characteristics	Negative	Positive	P value
	(n=1059, 57%)	(n=813, 43%)	
Female—no. (%)	606 (57)	509 (63)	0.002
Age—median (IQR)	30 (15–44)	36 (18–50)	0.004
Race/ethnicity—no. (%)*			0.003
Black/Pardo	813 (77)	671 (83)	
Others	245 (23)	142 (17)	
Income—no. (%)†			0.954
More than a minimum wage	409 (40)	276 (35)	
Equal or less than a minimum wage	388 (38)	357 (45)	
Without income	225 (22)	155 (20)	
BCG scar—no. (%)	950 (90)	721 (89)	0.498
HIV infection—no. (%)	32 (3)	14 (2)	0.097
Antiretroviral therapy—no. (%)	26 (8)	10 (7)	0.465
Education—no. (%)‡			0.137
Literate	943 (89)	743 (91)	
Illiterate	114 (11)	70 (9)	
Smoking—no. (%)§	253 (24)	246 (30)	0.002
Secondary smoking—no. (%)¶	319 (30)	281 (35)	0.04
Alcohol consumption—no. (%)**	565 (53)	442 (54)	0.674
Alcohol consumption (years)—median (IQR)	10 (3–18)	14 (5–25)	0.002
CAGE score of 2 and above—no. (%)††	110 (30)	67 (24)	0.075
Illicit drug use—no. (%)	102 (10)	84 (10)	0.640
Comorbidities—no. (%)‡‡	242 (23)	199 (245)	0.411
Diabetes	58 (6)	34 (4)	0.235
Hypertension	120 (11)	111 (14)	0.137

Data represent no. (%). or median with IQR.

29 (1.5%) individuals had an indeterminate result in the first or second IGRA.

*Race ethnicity was self-reported. Others ethnicities: white, Asian and Indian.

†Income: monthly money received in the household, categorised in wage on this study. One Brazilian minimum wage was US$266/month (The World Bank), the average value in the period (2015–2019).

‡Education: categorised in: literate: defined as patients who reported ability to read and write. Illiterate: defined as patients who reported unable to read or write.

§Smoking: defined as patients who reported currently smoking or before being diagnosed with tuberculosis.

¶Secondary smoking: patients who reported being exposed to cigarette smoke from other smokers (passive smoker).

**Alcohol consumption: defined as patients who reported consuming currently alcohol or before being diagnosed with tuberculosis.

††Alcohol abuse was defined as a CAGE score ≥2 points as described in Methods. Continuous variables were compared using the Mann-Whitney U test and categorical variables were using the Fisher’s exact test (2×2) or Pearson’s χ^2^ test. Missing information was identified in three variables: race (1 (0.05%)), income (62 (3.3%)) and education (2 (0.1%)). These three variables were imputed based on baseline variables and outcome.

‡‡Comorbidities: at least one comorbidity (diabetes, hypertension, cancer, chronic obstructive pulmonary/emphysema, kidney disease, heart disease, liver disease and depression).

### Losses of LTBI cascade of care

The losses in the LTBI cascade are presented in figure 1. We observed losses at all steps of the cascade. Most losses occurred early in the cascade, where 40% of the identified contacts were not evaluated for screening, and another 14% were not examined (figure 1A). Of the 933 contacts who met criteria for receiving TPT, 441 (47%) initiated TPT (figure 1B). Of the 441 who initiated treatment, 239 (54% of those initiating; 26% of those eligible; 6% of all contacts) completed it (figure 1A–C). Of those who completed the recommended treatment, all received isoniazid: 24 contacts for 6 months and 215 contacts for 9 months.

**Figure 2:**
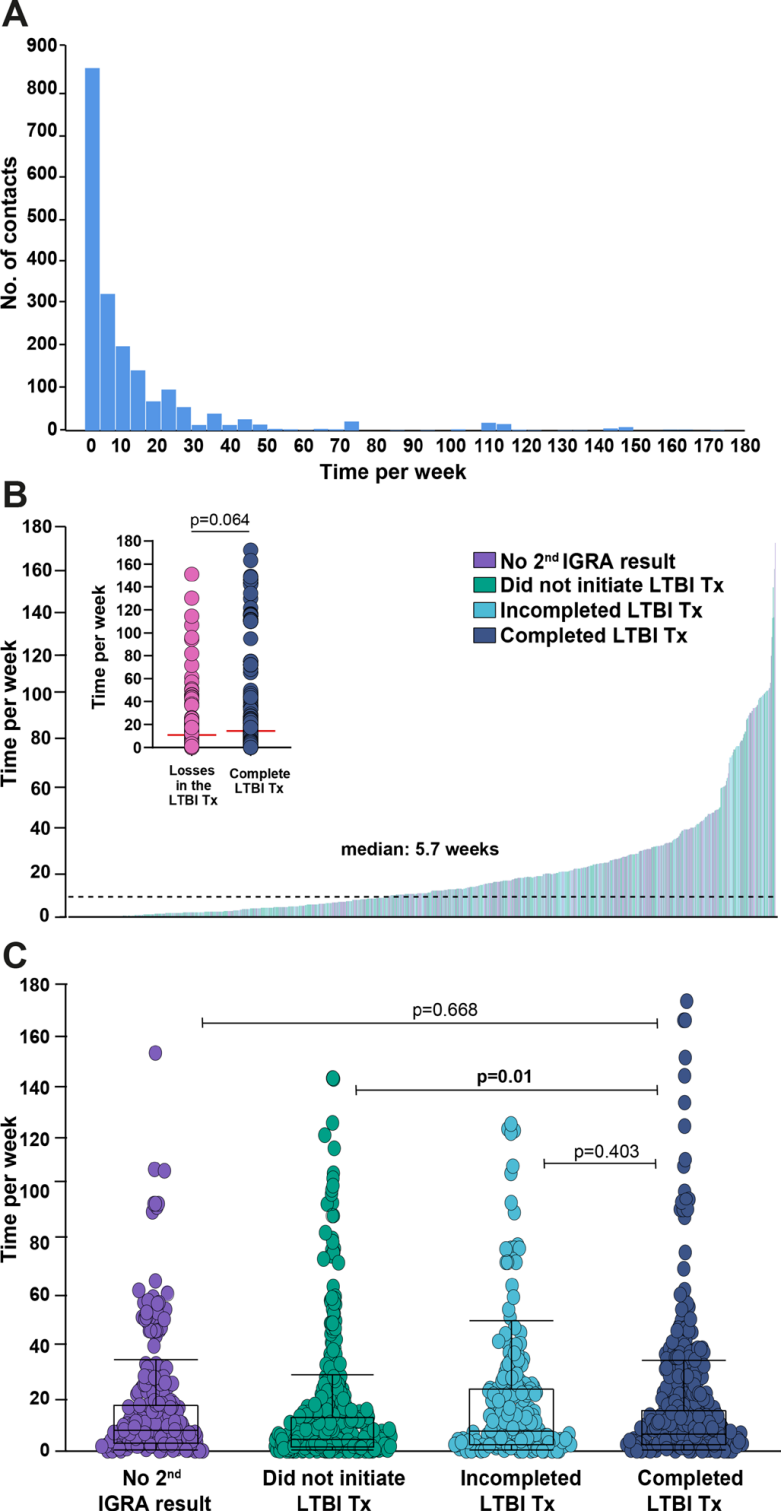
Time from detection of tuberculosis (TB) index cases to screening of their contacts. (A) Histograms representing the frequency of TB contacts between time (per week) from visit 1 of the TB index case and visit 1 of the contact. (B) Histograms representing time (per week) between visit 1 of the TB index case and visit 1 of the contact by type of losses in the latent tuberculosis infection (LTBI) cascade for care. (C) Scatter plots show each contact and case and the time difference between visit 1 of the TB index case and visit 1 of the contact by type of losses in the LTBI cascade for care. Did not perform second interferon-gamma release assay (IGRA; n=236), did not initiate recommended TB preventive therapy (TPT; n=493), did not complete TPT (n=202) and complete TPT (n=247).

Analyses of the time from detection of the PTB index cases to screening of contacts are presented in figure 2. Contacts were evaluated on average approximately 1 month after the diagnosis of the TB index case (median=36 days) (figure 2A–B). Of note, contacts with different outcomes in the LTBI cascade could not be distinguished based on time from identification of the TB index case and the first screening for LTBI (figure 2B–C).

### Factors associated with overall losses of LTBI cascade of care

**Figure 3:**
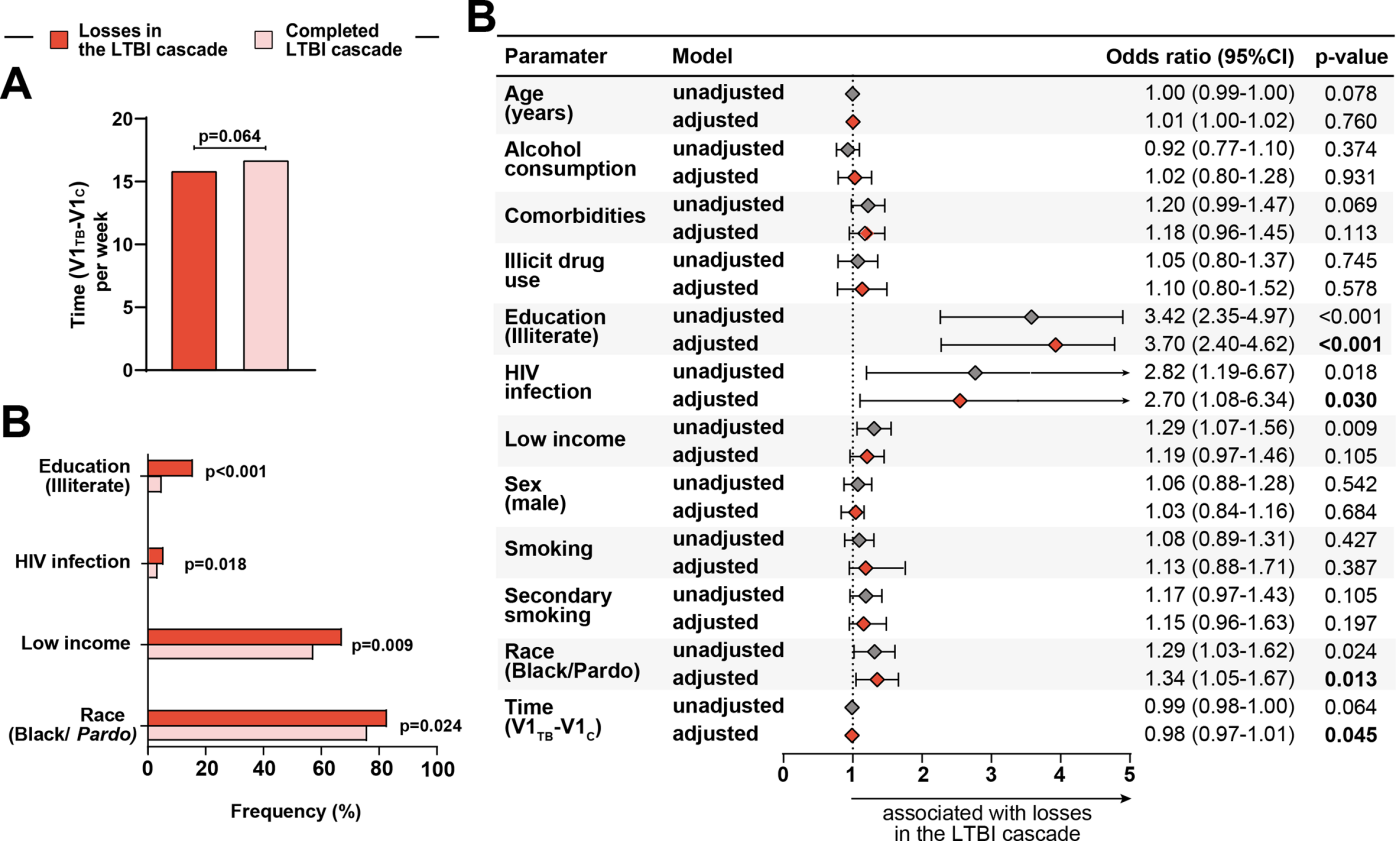
Association between epidemiological and clinical characteristics and losses in the latent tuberculosis infection (LTBI) cascade of care. (A) Time (per week) distribution among losses in the LTBI cascade of care and those who completed the LTBI cascade. Data were compared using the Mann-Whitney U test. (B) Frequency of race/ethnicity (black and *pardo*), income (see definition below), secondary smoking, comorbidities (see definition below) and education (illiterate) between TB contacts stratified based on losses in the LTBI cascade. Data were compared using Fisher’s exact test. (C) Generalised estimating equations analysis to evaluate association between epidemiological and clinical characteristics and losses in the LTBI cascade of care. The study population was stratified according to complete TB preventive therapy (TPT) in the LTBI cascade (complete LTBI cascade of care vs losses in the LTBI cascade of care, see online supplemental table 1 for detailed univariate comparisons). A multivariable analysis (see the Methods section for details) was employed with each variable individually (unadjusted) and variables (panels A and C) were included in a multivariable model (adjusted). In all the comparisons, significant p values are shown in bold-type font. Comorbidities: at least one comorbidity (diabetes, hypertension, cancer, chronic obstructive pulmonary/emphysema, kidney disease, heart disease, liver disease and depression). Low income: without income/equal or less than a minimum wage (reference: more than a minimum wage) ace (Black/*Pardo*) reference: white, Asian, Indian. Ethnicity was self-reported. Time (V1_TB_-V1_C_), time (in weeks) difference between the visit 1 of the tuberculosis case and the visit 1 of the contact.

Figure 3 presents associations between clinical and demographic factors and overall losses in the LTBI cascade of care (ie, all losses in the LTBI cascade combined). There were no significant differences in sex distribution or in the ages of patients who were lost in the LTBI cascade or completed treatment. However, in univariate analyses, illiteracy (unadjusted OR (unOR): 3.42, 95% CI 2.35 to 4.97, p<0.001), HIV infection (unOR: 2.82, 95% CI 1.19 to 6.67, p=0.018) and wage <the minimum established for Brazil (unOR: 1.29, 95% CI 1.07 to 1.56, p=0.009) and black or pardo (unOR: 1.29, 95% CI 1.03 to 1.62, p=0.024) were associated with losses in the LTBI cascade of care (online supplemental table 1, figure 3B). Multivariable analyses also revealed that illiteracy (adjusted OR (aOR): 3.67, 95% CI (CI) 2.40 to 5.62, p>0.001), HIV infection (aOR: 2.63, 95% CI 1.08 to 6.4, p=0.032) and race black or pardo (aOR: 1.33, 95% CI 1.06 to 1.67, p=0.014) were independently associated with losses in the LTBI cascade (figure 3B). The impact of time from the identification of the TB index case to the initial screening of the close contact on the overall losses in the LTBI cascade was only marginal, but statistically significant (figure 3B). Indeed, each increase in 1 day between the PTB diagnosis and the enrolment of the close contact resulted in an adjusted odds of 0.99 (95% CI 0.98 to 1.0, p=0.032) of being lost in the LTBI cascade of care.

10.1136/bmjgh-2021-005969.supp1 Supplementary data

### Factors associated with losses at specific stages of the LTBI cascade of care

Considering those participants who were recommended to take TPT but did not initiate (492 of 933; 53%) (figure 1B), they had a lower individual income (p=0.005), a shorter time between the diagnosis of the TB index case and evaluation of the contact (p=0.027), a higher rate of illiteracy (p<0.001) and a higher rate of secondary smoking (p=0.001). Three of these four variables were independently associated with not initiating TPT: lower income (aOR: 1.36, 95% CI 1.03 to 1.80, p=0.032), illiteracy (aOR: 3.05, 95% CI 1.92 to 4.86, p<0.001) and secondary smoking (aOR: 1.61, 95% CI 1.19 to 2.16, p=0.002; online supplemental figure 2C) and online supplemental table 2).

Of 454 contacts who initiated TPT, 202 (44%) did not complete TPT (figure 1A and online supplemental table 3). Those who did not complete TPT were younger (p=0.004) and more likely to be black or *pardo* (p=0.012) than contacts who completed TPT (online supplemental table 3). Finally, race black or *pardo* (aOR: 1.71, 95% CI 1.01 to 2.90, p=0.048) and age (aOR: 0.98, 95% CI 0.97 to 1.00, p=0.009) were associated with non-completion of TPT after adjusting for confounding variables (online supplemental figure 6 and online supplemental table 4).

## Discussion

In this prospective, multicentre, cohort study, we analysed the cascade of care of LTBI in Brazil and found that HIV infection, illiteracy, low income and race black or *pardo* were independently associated with cumulative losses at cascade. All of these factors are related to low socioeconomic status (SES). SES is one of the main determinants of health, with people from a lower SES having a lower life expectancy. Several serious conditions are associated with low SES, such as hypertension, diabetes and depression.^18^ In addition, low SES is associated with a higher burden of prevalent HIV infection and poorer HIV treatment outcomes.^19 20^ Our findings highlight the relevance of social factors in TB care, previously noted in different studies.^21^ These results reinforce the need to consider social determinants when developing healthcare public policies.

We found losses in all steps of the cascade, but the most substantial loss occurred before the visit to the clinic, when individuals identified by the TB index cases as contacts did not come for evaluation. This is consistent with findings from previous studies, where with greater losses included completion of testing in between of people intended for screening.^5^ Two main reasons for this finding have been identified previously: (1) not having interest in being tested and (2) self-perceived low risk of TB infection.^5^ In a study conducted in Myanmar, of 1908 MDR-TB contacts, some participants refused the contact investigation because they strongly believed that they did not have TB disease given that they did not have any signs or symptoms.^22^

Interestingly, the factors associated with loss in the LTBI care cascade are also factors associated with increased TB risk (factors that impair the host’s defence against TB infection and disease, such as HIV infection and low SES).^21^

LTBI was significantly more common in black/*pardo* individuals. There is still not enough evidence on the relationship between ethnicity and race associated with complete treatment for ILTB. However, Viana *et al* 23 have showed racial inequalities in incomplete anti-TB treatment, with the black race presenting with the highest frequency of non-compliance (10.5%). This could be explained by the more precarious living conditions, with lower incomes and with limitations in healthcare, more commonly observed in the black and *pardo* population.^24^ These findings of race differences in LTBI merit additional investigation; they could reflect social dynamics in Brazil.

A recently published meta-analysis^25^ and literature review analysing evidence on interventions to reduce loss in LTBI cascade found that completion of the initial assessment (eg, return for medical visits) is significantly improved by financial and non-financial incentives. To a lesser extent, home visits, reminders and healthcare worker education were also shown to be helpful. Incentives and education of individuals who are indicated for LTBI improved treatment acceptance rates.

Another point worth noting in our study was that less than half of TB contacts who had an indication for TPT attended the medical visit and started it. This was lower than what was found in a meta-analysis (72.2%, 95% CI: 48% to 96%) of 6 cohorts from countries at the low-income or middle-income level.^5^ However, our study included patients who came to the medical visit and refused treatment, as well as patients who never came to get the test results and were not seen by the physician to have the issue approached.

In our study, 54% of TB contacts who initiated TPT completed it. We found that lower income was associated with non-complete TPT. A similar proportion (56%) was observed in a cohort of 336 TB contacts in a primary healthcare service in São Paulo state, Brazil and in studies in low or middle-income countries (about 50%).^5 7^ Barriers to treatment completion demonstrated before are diverse and vary according to location. Common barriers include side-effects to drugs, long treatment duration, issues related to the health system and individual concerns, such as substance use.^5^ Financial barriers and low level of knowledge about the cost–benefit of treatment have also been associated with poor treatment adherence in LTBI patients.^26^

Our study had several limitations. Because the contacts were enrolled in a study conducted in referral centres, and under research conditions, the results may not be generalisable to all close contacts of TB index cases. Second, the contacts were of culture-confirmed TB cases who also enrolled into the RePORT-Brazil observational cohort. This raises the question of representativeness of the study cohort, and generalisability of the findings. Although the RePORT-Brazil cohort of TB cases is representative of all patients with TB in Brazil,^11^ it is unclear if the close contacts are representative of all close contacts in Brazil. Third, the low percentage of contacts with HIV and illiteracy in the study population could have led to possibly less precise estimates. Fourth, participants lost to follow-up could have completed TPT elsewhere.

With our limitations noted as above, the results presented here illuminate substantial losses to follow-up among a population at high risk of developing active TB disease. Mitigating losses in the LTBI care cascade and the factors associated is an important step for the control and eradication of TB.

## Data Availability

Data are available upon reasonable request. Data Availability Statement: Due to ethical restrictions regarding participant's privacy, data are available upon request. Data are available upon request for researchers who meet the criteria for access to confidential data. Additional requests for the data may be sent to the corresponding author Marcelo Cordeiro-Santos (marcelocordeiro.br@gmail.com).
