# Supplementary material for: Determinants of losses in the latent tuberculosis infection cascade of care in Brazil
Source: BMJ Glob Health. 2021 Sep 9;6(9):e005969. doi: 10.1136/bmjgh-2021-005969 (PMC8438757; doi:10.1136/bmjgh-2021-005969)
Supplement: Supplementary data [file bmjgh-2021-005969supp002.pdf]

Supplementary Methods

Description of sites from the Regional Prospective Observational Research in Tuberculosis (RePORT)-Brazil

Salvador (BA) site:

Unit of the José Silveira Foundation (FJS), a non-profit institution, the Brazilian Tuberculosis Research Institute (IBIT) in the state of Bahia, Brazil and accredited by the government as a TB treatment center. Currently, FJS / IBIT serves as a reference center for primary TB care and annually treats 10 to 15% of TB cases in the city of Salvador. This Institute is part of the National TB Control Program and follows all guidelines and recommendations from the Brazilian Society of Pulmonology and Tisiology, which are similar to World Health Organization recommendations.

Manaus (AM) site:

The Fundação de Medicina Tropical (FMT) Dr. Heitor Vieira Dourado (Amazonas) is the leading institution of Tropical Diseases in the Amazonas State. FMT is a referral center for the diagnosis and treatment of infectious (including tuberculosis), parasitic and dermatological diseases, admitting in their facilities patients referred by the health network of Manaus and from the countryside of Amazonas State. The FMT-HVD Outpatient Clinic has 40 medical offices and held more than 285,000 health consultations in 2016.

Rio de Janeiro (RJ) sites:

The Instituto Nacional de Infectologia Evandro Chagas (INI) Hospital is a reference hospital within the Oswaldo Cruz Foundation (Fiocruz) The outpatient unit provides care to patients referred for tuberculosis, acute febrile diseases, and HIV/AIDS, among others. INI has extensive laboratory capabilities for infectious diseases, including TB. The laboratory unit is comprised of hematology, biochemistry, mycology, virology, immunology, and microbiology laboratories.

Secretaria Municipal de Saúde do Rio de Janeiro e Clínica da Família Rinaldo Delamare, Rocinha is a low-income community of 70,000 inhabitants located in southern Rio de Janeiro, where TB incidence rates have reached levels four times higher than those recorded in the municipality. The population of Rocinha is 100% covered by the Brazilian primary health care program and has access to TB care in their community. The TB research site receives patients from the 3 clinics in Rocinha.

Tuberculosis Control Program – Secretaria de Saúde de Duque de Caxias. This center initially began in 1971 as a State Health Unit, but due to the high patient volume, it is now dedicated solely to the city of Duque de Caxias. This TB center accepts patients and referrals from other hospitals (primarily the Moacyr do Carmo Municipal Hospital and Adam Pereira Nunes Hospital), Emergency Care Units, Family Health Programs, and primary units of the municipality. A Tuberculosis Clinical Research Laboratory was founded at the Municipal Health Secretariat of Duque de Caxias in 2014, through technical and scientific cooperation between the Duque de Caxias Municipal Health Secretariat and the Federal University of Rio de Janeiro (UFRJ).

For more information: https://reportbrazil.org/

Description inclusion and exclusion criteria for TB cases enrolled in RePORT-Brazil

Inclusion Criteria:

- Persons aged ≥ 18 years (or children enrolled in Cohort B who develop active TB) who have agreed to participate in the study and sign an Informed Consent Form.
- Pulmonary TB with culture-positive sputum (with or without a positive AFB smear or Xpert® MTB/ RIF assay). Persons who have signs and symptoms of TB and/or a positive AFB smear or Xpert® MTB/RIF assay or a chest x-ray consistent with TB are eligible, but they must have a positive AFB culture to remain in the study*.
- New or recurrent TB.
- Willingness to undergo HIV testing if there is no documented positive HIV test.

*Children enrolled in Cohort B who develop active TB do not necessarily need a positive AFB culture to remain in the study.

Exclusion Criteria

- Persons currently receiving anti-TB therapy (defined as > 7 days of appropriate TB treatment in the last 30 days).
- Have received more than seven days of fluoroquinolone antibiotic therapy, for any reason, within the last 30 days.
- Women who are pregnant or breastfeeding*.

Not remaining in the region during the study period/or having plans to move away.

*Women who enroll but become pregnant after enrollment will remain in the study. However, due to concerns about blood volumes, blood will not be drawn from pregnant women.
