# Supplementary material for: Determinants of losses in the latent tuberculosis infection cascade of care in Brazil
Source: BMJ Glob Health. 2021 Sep 9;6(9):e005969. doi: 10.1136/bmjgh-2021-005969 (PMC8438757; doi:10.1136/bmjgh-2021-005969)
Supplement: Supplementary data [file bmjgh-2021-005969supp001.pdf]

Supplementary Material

Content:

1. **Supplementary Table 1**
2. **Supplementary Table 2**
3. **Supplementary Table 3**
4. **Supplementary Table 4**
5. **Supplementary Figure 1**
6. **Supplementary Figure 2**
7. **Supplementary Figure 3**
8. **Supplementary Figure 4**

Supplementary Tables

Supplementary Table 1. Characteristics of contacts according to losses and completeness of LTBI cascade of care

Characteristics	Lost in the cascade (n=895)*	Completed the cascade (n=1001)	p-value
**Female– no. (%)**	538 (60)	589 (59)	0.542
**Age – median (IQR)**	31 (16-47)	33 (17-47)	0.078
**Race/Ethnicity – no. (%)**			0.024
Black/ Pardo	741 (83)	761 (76)	
Others	153 (17)	240 (24)	
**Income – no. (%)**			0.009
More than a minimum wage	285 (33)	412 (43)	
Equal or less than a minimum wage	386 (44)	365 (38)	
Without income	197 (23)	189 (19)	
**BCG scar – no. (%)**	802 (90)	893 (89)	0.820
**Time ($V1_{TB}$-$V1_{C}$) – median (IQR)**	33 (8-82)	41 (14-102)	0.055
**HIV infection – no. (%)**	31 (4)	15 (2)	0.018
**Antiretroviral therapy – no. (%)**	23 (74)	13 (87)	0.460
**Education– no. (%)**			<0.001
Literate	753 (84)	950 (95)	
Illiterate	141 (16)	50 (5)	

BMJ Publishing Group Limited (BMJ) disclaims all liability and responsibility arising from any reliance placed on this supplemental material which has been supplied by the author(s)

**Smoking – no. (%)**	247 (28)	254 (25)	0.427
**Secondary smoking – no. (%)**	319 (36)	284 (28)	0.105
**Alcohol consumption – no. (%)**	471 (53)	545 (54)	0.433
**Alcohol consumption (years)-median (IQR)**	12 (4-22)	11 (3-20)	0.337
**CAGE score of 2 and above [a] – no. (%)**	82 (26)	96 (29)	0.333
**Illicit drug use – no. (%)**	91 (10)	97 (10)	0.758
**Comorbidities[b]– no. (%)**	241 (27)	204 (20)	0.069
Diabetes	43 (5)	52 (5)	0.752
Hypertension	132 (15)	101 (10)	0.003

Table note: Data represent no. (%), except for age time, which is presented as median and interquartile range (IQR) Continuous variables were compared using the Mann-Whitney *U* test and categorical variables were using the Fisher's exact test (2x2) or Pearson's chi-square test. Time ($V1_{TB}$-$V1_{C}$): time (in weeks) difference between the visit 1 of the TB case and the visit 1 of the contact.

[*]Five contacts are ongoing in the TPT

[a]Alcohol abuse was defined as a CAGE score ≥ 2 points *(Ewing, J.A. 1984. Detecting alcoholism: The CAGE questionnaire. JAMA).*

[b] Comorbidities: At least one comorbidity (diabetes, hypertension, cancer, chronic obstructive pulmonary /emphysema, kidney disease, heart disease, liver disease and depression)

Abbreviations: TB: tuberculosis. LTBI: latent tuberculosis infection. TPT: TB preventive therapy

Supplementary Table 2. Characteristics of contacts according to Initiation of recommended TPT

Characteristics	No initiation of recommended TPT (n=492)	Initiation of recommended TPT (n=454*)	p-value
**Female– no. (%)**	299 (61)	272 (60)	
**Age – median (IQR)**	32.5 (11-48)	32.6 (15-48)	0.902
**Race/Ethnicity – no. (%)**			0.247
Black/ Pardo	408 (83)	362 (80)	
Others	83 (17)	92 (20)	
**Income – no. (%)**			0.005
More than a minimum wage	150 (31)	171 (39)	
Equal or less than a minimum wage	236 (49)	175 (40)	
Without income	94 (20)	92 (21)	
**BCG scar – no. (%)**	444 (90)	400 (88)	0.296
**Time ($V1_{TB}$-$V1_{C}$) – median (IQR)**	24 (7-71)	45 (15-102)	0.027
**HIV infection – no. (%)**	19 (4)	26 (6)	0.555
**Antiretroviral therapy – no. (%)**	12 (63)	23 (89)	0.070
**Education– no. (%)**			<0.001
Literate	379 (77)	404 (89)	

Illiterate	113 (23)	50 (11)	
**Smoking – no. (%)**	131 (27)	134 (30)	0.911
**Secondary smoking – no. (%)**	190 (39)	134 (30)	0.001
**Alcohol consumption – no. (%)**	233 (47)	233 (51)	0.241
**Alcohol consumption (years)- median (IQR)**	14 (5-21)	13 (4-23)	0.918
**CAGE score of 2 and above [a] – no. (%)**	31 (20)	40 (29)	0.133
**Illicit drug use – no. (%)**	37 (8)	54 (12)	0.128
**Comorbidities [b]– no. (%)**	128 (26)	102 (23)	0.295
Diabetes	23 (5)	20 (4)	0.880
Hypertension	65 (13)	64 (14)	0.705

Table note: Data represent no. (%), except for age and time, which is presented as median and interquartile range (IQR) Continuous variables were compared using the Mann-Whitney *U* test and categorical variables were using the Fisher's exact test (2x2) or Pearson's chi-square test. Time ($V1_{TB}$-$V1_{C}$): time (in weeks) difference between the visit 1 of the TB case and the visit 1 of the contact.
* Among those to whom TPT was recommended (n=933)
[a] Alcohol abuse was defined as a CAGE score ≥ 2 points as described in Methods.
[b] Comorbidities: At least one comorbidity (diabetes, hypertension, cancer, chronic obstructive pulmonary /emphysema, kidney disease, heart disease, liver disease and depression)
Abbreviations: TB: tuberculosis. TPT: TB preventive therapy

Supplementary Table 3. Characteristics of contacts according to complete TB preventive therapy

Characteristics	Incomplete TPT (n=202*)	Complete TPT (n=247)	p-value
**Female– no. (%)**	116 (57)	153 (62)	0.470
**Age – median (IQR)**	28.2 (15-43)	37.5 (15-53)	0.004
**Race/Ethnicity – no. (%)**			0.012
Black/ Pardo	172 (85)	185 (75)	
Others	30 (15)	62 (25)	
**Income – no. (%)**			0.030
More than a minimum wage	66 (34)	105 (44)	
Equal or less than a minimum wage	82 (42)	92 (39)	
Without income	47 (24)	41 (17)	
**BCG scar – no. (%)**	176 (87)	219 (89)	0.663
**Time ($V1_{TB}$-$V1_{C}$) – median (IQR)**	45 (14-92)	47 (17-106)	0.217
**HIV infection – no. (%)**	12 (6)	14 (6)	0.361
**Antiretroviral therapy – no. (%)**	11 (92)	12 (86)	1.000
**Education– no. (%)**			0.982
Literate	176 (87)	224 (91)	
Illiterate	26 (13)	23 (9)	
**Smoking – no. (%)**	55 (27)	77 (31)	0.195

**Secondary smoking – no. (%)**	63 (31)	69 (28)	0.641
**Alcohol consumption – no. (%)**	111 (55)	119 (48)	0.156
**Alcohol consumption (years)- median (IQR)**	12 (3-24)	14 (4-23)	0.581
**CAGE score of 2 and above [a] – no. (%)**	26 (34)	12 (19)	0.058
**Illicit drug use – no. (%)**	27 (13)	25 (10)	0.876
**Comorbidities[b]– no. (%)**	55 (27)	47 (19)	0.119
Diabetes	6 (3)	14 (6)	0.250
Hypertension	35 (17)	29 (12)	0.104

Table note: Data represent no. (%), except for age and time, which is presented as median and interquartile range (IQR) Continuous variables were compared using the Mann-Whitney *U* test and categorical variables were using the Fisher's exact test (2x2) or Pearson’s chi-square test. Time ($V1_{TB}$-$V1_{C}$): time (in weeks) difference between the visit 1 of the TB case and the visit 1 of the contact.

*4 no recommended

[a] Alcohol abuse was defined as a CAGE score ≥ 2 points as described in Methods

[b] Comorbidities: At least one comorbidity (diabetes, hypertension, cancer, chronic obstructive pulmonary /emphysema, kidney disease, heart disease, liver disease and depression)

Abbreviations: TB: tuberculosis. LTBI: latent tuberculosis infection. TPT: TB preventive therapy

Supplementary Table 4. Characteristics of contacts according to 2nd IGRA performed

**Characteristics**	**2nd IGRA not performed (n=242)**	**2nd IGRA performed (n= 967)**	**p-value**
**Female– no. (%)**	142 (59)	548 (57)	0.836
**Age – median (IQR)**	27.1 (16-44.)	30.3 (15-45)	0.412
**Race/Ethnicity – no. (%)**			0.883
Black/ Pardo	193 (80)	749 (78)	
Others	48 (20)	218 (23)	
**Income – no. (%)**			0.048
More than a minimum wage	81 (35)	392 (42)	
Equal or less than a minimum wage	83 (36)	362 (39)	
Without income	69 (29)	182 (19)	
**BCG scar – no. (%)**	221 (91)	865 (90)	0.475
**Time ($V1_{TB}$-$V1_{C}$) – median (IQR)**	36 (15-96)	40 (13-99)	0.955
**HIV infection – no. (%)**	7 (3)	28 (3)	0.883
**Antiretroviral therapy – no. (%)**	5 (71)	24 (86)	0.576
**Education– no. (%)**			0.632
Literate	211 (88)	860 (89)	

Illiterate	30 (12)	106 (11)	
**Smoking – no. (%)**	66 (27)	227 (24)	0.301
**Secondary smoking – no. (%)**	76 (32)	284 (30)	0.872
**Alcohol consumption – no. (%)**	133 (55	510 (53)	0.565
**Alcohol consumption (years)- median (IQR)**	10 (2-18)	10 (3-18)	0.644
**CAGE score of 2 and above [a] – no. (%)**	26 (27)	101 (32)	0.448
**Illicit drug use – no. (%)**	29 (12)	91 (9)	0.231
**Comorbidities[b]– no. (%)**	73 (30)	196 (20)	0.023
Diabetes	19 (8)	43 (4)	0.049
Hypertension	40 (17)	93 (10)	0.004

Table note: Data represent no. (%), except for age and time, which is presented as median and interquartile range (IQR). Continuous variables were compared using the Mann-Whitney *U* test and categorical variables were using the Fisher's exact test (2x2) or Pearson's chi-square test. Time ($V1_{TB}$-$V1_{C}$): time (in weeks) difference between the visit 1 of the TB case and the visit 1 of the contact.

[a] Alcohol abuse was defined as a CAGE score ≥ 2 points as described in Methods.

[b] Comorbidities: At least one comorbidity (diabetes, hypertension, cancer, chronic obstructive pulmonary /emphysema, kidney disease, heart disease, liver disease and depression)

Abbreviations: TB: tuberculosis; IGRA: interferon-gamma release assay.

Supplementary Figures

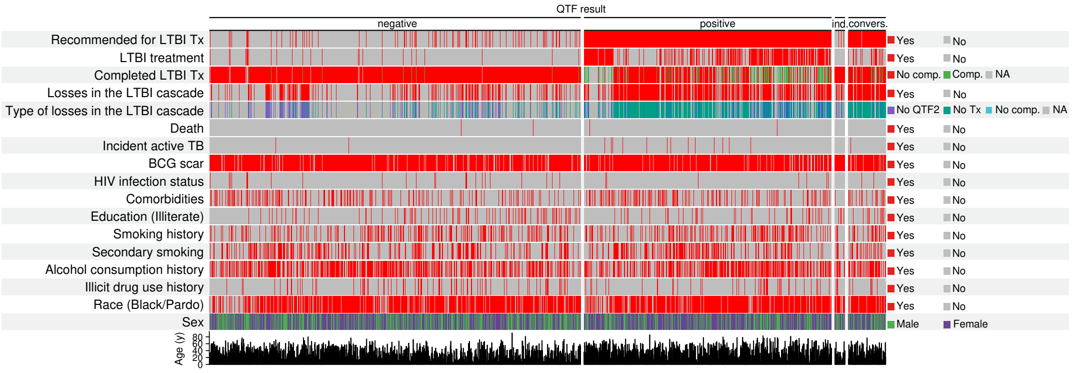

Supplementary Figure 1. Characteristics of study participants

Color map based on TB contacts grouped according to final IGRA results shows the overall characteristics of the study participants. A hierarchical clustering analysis (Ward's method) was employed to group individuals based on the overall profile of each study participant which each IGRA subgroup.

Alcohol abuse was defined as a CAGE score ≥ 2 points. Comorbidities: At least one comorbidity (diabetes, hypertension, cancer, chronic obstructive pulmonary /emphysema, kidney disease, heart disease, liver disease and depression

Abbreviations: IGRA: interferon-gamma release assay, Ind: Indeterminate, Convers: conversion, No IGRA 2: Did not perform 2nd IGRA, LTBI: latent tuberculosis infection, TB: tuberculosis. TPT: TB preventive therapy.

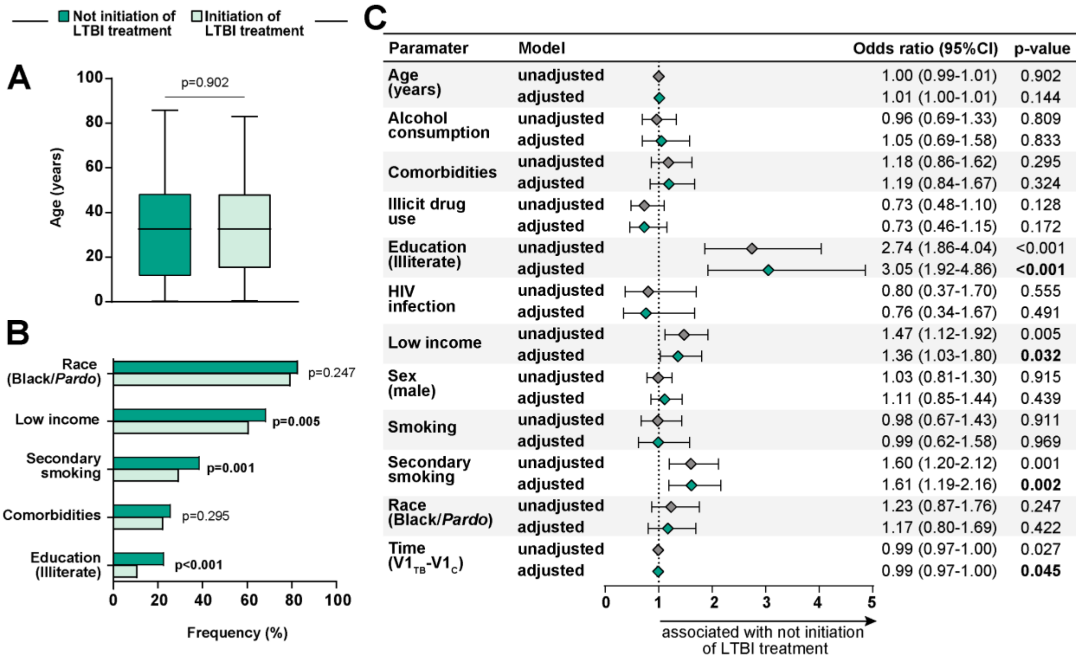

Supplementary Figure 2. Association between epidemiological and clinical characteristics and initiation of TPT

(A) Age distribution among TB contacts who did not initiate TPT and those who initiated the TPT. Data were compared using the Mann-Whitney *U* test. **(B)** Frequency of Race/Ethnicity (black and *pardo*), income (see definition below), secondary smoking, comorbidities (see definition below) and education (illiterate) between TB contacts stratified based on initiation of TPT**.** Data were compared using Fisher's exact test. **(C)** Generalized estimating equations analysis to evaluate association between epidemiological and clinical characteristics and not initiation of TPT**.** The study population was stratified according to initiation of TPT (not initiation of TPT and initiation of the TPT, see Supplementary Table 2 for detailed univariate comparisons). A multivariable analysis (see Methods for details) was employed with each variable individually (unadjusted) and variables (Panels A and **C)** were included in a multivariable model (adjusted). In all the comparisons, significant p-values are shown in bold-type font.

Comorbidities: At least one comorbidity (diabetes, hypertension, cancer, chronic obstructive pulmonary /emphysema, kidney disease, heart disease, liver disease and depression).

Low income: without income/equal or less than a minimum wage (reference: more than a minimum wage)

Race (Black/*Pardo*) reference: White, Asian, Indian.

Abbreviations: 95%CI: 95% confidence interval. Time (V1TB-V1C): time (in weeks) difference between the visit 1 of the TB case and the visit 1 of the contact. TPT: TB preventive therapy.

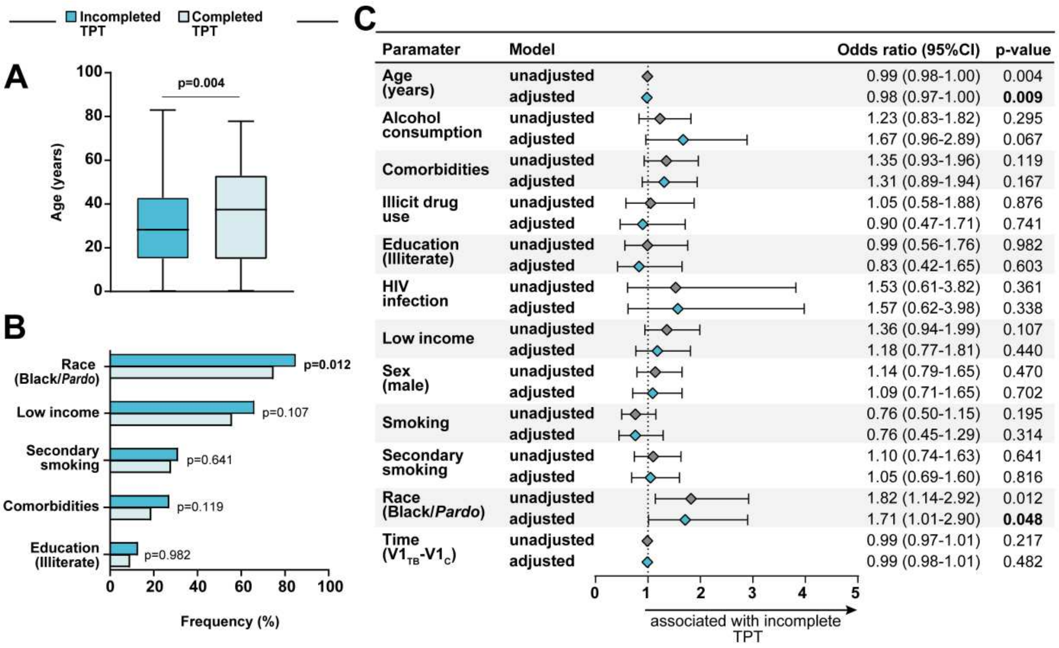

Supplementary Figure 3. Association between epidemiological and clinical characteristics and completeness of the TB preventive therapy

(A) Age distribution among those who did not complete the TPT and who complete the TPT. Data were compared using the Mann-Whitney *U* test. **(B)** Frequency of Race/Ethnicity (black and *pardo*), income (see definition below), secondary smoking, comorbidities (see definition below) and education (illiterate) between TB contacts stratified based completeness of the TPT (complete vs. incomplete)**.** Data were compared using Fisher's exact test. **(C)** Generalized estimating equations analysis to evaluate association between epidemiological and clinical characteristics and who did not complete the TPT and who complete the TPT. The study population was stratified according to complete TPT in the LTBI cascade (incomplete TPT vs. complete cascade of care, see Supplementary Table 3 for detailed univariate comparisons). A multivariable analysis (see Methods for details) was employed with each variable individually (unadjusted) and variables (panels A

and **C)** were included in a multivariable model (adjusted). In all the comparisons, significant p-values are shown in bold-type font.

Comorbidities: At least one comorbidity (diabetes, hypertension, cancer, chronic obstructive pulmonary /emphysema, kidney disease, heart disease, liver disease and depression).

Low income: without income/equal or less than a minimum wage (reference: more than a minimum wage)

Race (Black/*Pardo*) reference: White, Asian, Indian.

Abbreviations: 95%CI: 95% confidence interval. Time ($V1_{TB}$-$V1_{C}$): time (in weeks) difference between the visit 1 of the TB case and the visit 1 of the contact. TPT: TB preventive therapy.

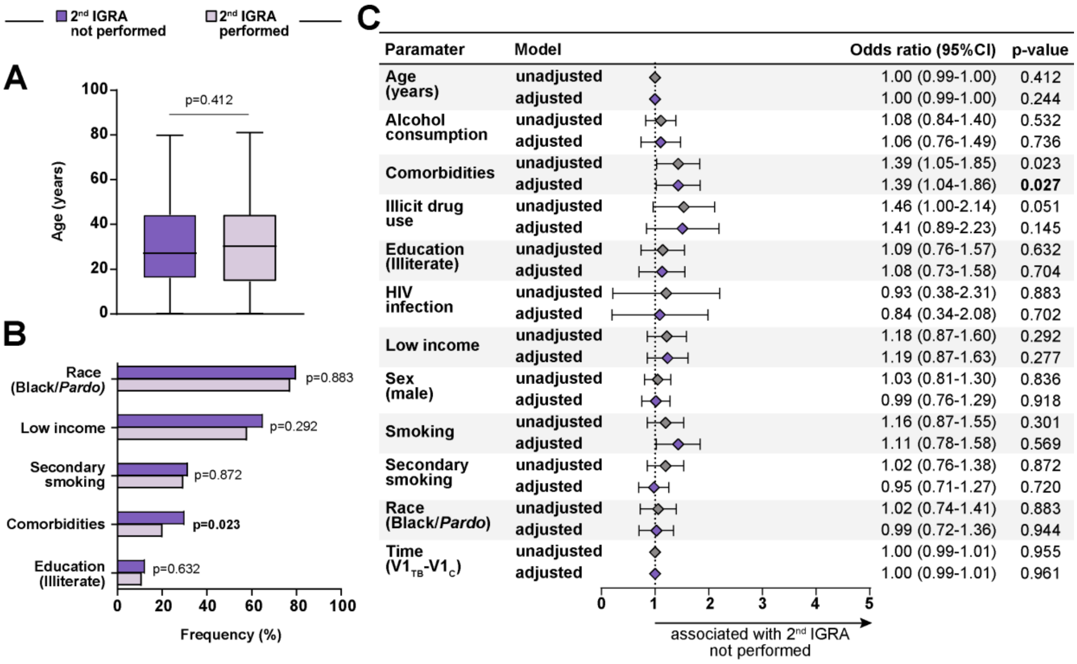

Supplementary Figure 4. Association between epidemiological and clinical characteristics and performing the 2nd IGRA test at month 6 when indicated. **(A)** Age distribution among those who performed the 2nd IGRA and those not performed the 2nd IGRA. Data were compared using the Mann-Whitney *U* test. **(B)** Frequency of Race/Ethnicity (black and *pardo*), income (see definition below), secondary smoking, comorbidities (see definition below) and education (illiterate) between TB contacts stratified based on performing the 2nd IGRA**.** Data were compared using the Fisher's exact test. **(C)** Generalized estimating equations analysis to evaluate association between epidemiological and clinical characteristics and losses in the LTBI cascade of care**.** The study population was stratified according to perform 2nd IGRA (2nd IGRA not performed and 2nd IGRA performed, see Supplementary Table 3 for detailed univariate comparisons). A multivariable analysis (see Methods for details) was employed with each variable individually (unadjusted) and variables (panels A and C) were included in a

multivariable model (adjusted). In all the comparisons, significant p-values are shown in bold-type font.

Comorbidities: At least one comorbidity (diabetes, hypertension, cancer, chronic obstructive pulmonary /emphysema, kidney disease, heart disease, liver disease and depression).

Low income: without income/equal or less than a minimum wage (reference: more than a minimum wage)

Race (Black/*Pardo*) reference: White, Asian, Indian.

Abbreviations: 95%CI: 95% confidence interval. Time (V1TB-V1C): time (in weeks) difference between the visit 1 of the TB case and the visit 1 of the contact.
